# Evaluating statistical analysis models for RNA sequencing experiments

**DOI:** 10.3389/fgene.2013.00178

**Published:** 2013-09-17

**Authors:** Pablo D. Reeb, Juan P. Steibel

**Affiliations:** ^1^Department of Fisheries and Wildlife, Michigan State UniversityEast Lansing, MI, USA; ^2^Department of Statistics, Universidad Nacional del ComahueCinco Saltos, Argentina; ^3^Department of Animal Science, Michigan State UniversityEast Lansing, MI, USA

**Keywords:** RNA-seq, plasmodes, simulation, type I error, linear models

## Abstract

Validating statistical analysis methods for RNA sequencing (RNA-seq) experiments is a complex task. Researchers often find themselves having to decide between competing models or assessing the reliability of results obtained with a designated analysis program. Computer simulation has been the most frequently used procedure to verify the adequacy of a model. However, datasets generated by simulations depend on the parameterization and the assumptions of the selected model. Moreover, such datasets may constitute a partial representation of reality as the complexity or RNA-seq data is hard to mimic. We present the use of plasmode datasets to complement the evaluation of statistical models for RNA-seq data. A plasmode is a dataset obtained from experimental data but for which come truth is known. Using a set of simulated scenarios of technical and biological replicates, and public available datasets, we illustrate how to design algorithms to construct plasmodes under different experimental conditions. We contrast results from two types of methods for RNA-seq: (1) models based on negative binomial distribution (edgeR and DESeq), and (2) Gaussian models applied after transformation of data (MAANOVA). Results emphasize the fact that deciding what method to use may be experiment-specific due to the unknown distributions of expression levels. Plasmodes may contribute to choose which method to apply by using a similar pre-existing dataset. The promising results obtained from this approach, emphasize the need of promoting and improving systematic data sharing across the research community to facilitate plasmode building. Although we illustrate the use of plasmode for comparing differential expression analysis models, the flexibility of plasmode construction allows comparing upstream analysis, as normalization procedures or alignment pipelines, as well.

## Introduction

RNA sequencing (RNA-seq) technology is being rapidly adopted as the platform of choice for high-throughput gene expression analysis (Ozsolak and Milos, 2011). Many methods have been proposed to model relative transcript abundances obtained in RNA-seq experiments but it is still difficult to evaluate whether they provide accurate estimations and inferences.

Sound statistical analysis of RNA-seq data should consider not only the factors of any basic experimental design, but also the characteristics of “omic” studies (genomic, proteomic, transcriptomic, etc.). An RNA-seq experimental design must consider treatment and block structures, and combine them according to the principles of a well-planned design: randomization, blocking, and replication (Auer and Doerge, 2010). Typically, fixed or random effects such as library multiplexing, sequencing lane, flow cell, individual sample, tissue, or time can be crossed or nested with treatments or other experimental conditions. Such a design is used to model thousands of correlated variables (i.e., transcripts), usually, in a context of small number of biological replicates. Although the development of reliable models that account for all these factors is challenging, it is even more difficult to assess the validity of a particular analysis model (Pachter, 2011).

Validity of statistical models for differential expression analyses has been evaluated by (1) applying the model to a novel dataset, (2) deriving analytical proofs, (3) using simulations, (4) comparing to a gold-standard measure, or (5) constructing plasmodes. In (1) the true status of nature is unknown, therefore this method must only be accepted as an illustration and not as evidence to support a model. However, any of the last four options, or a combination of them, could be used to demonstrate adequacy of a model. Obtaining a mathematical demonstration (2), may be impossible for some models (Gadbury et al., 2008). Most of the models rely on assumptions that are difficult to verify and the consequences of departures from assumptions may not be clear. Computer simulation (3) has been the most commonly used procedure (Anders and Huber, 2010; McCarthy et al., 2012). This preference is due to easiness in creating datasets under diverse scenarios by controlling the set of parameters used in the simulation. Nevertheless, such generated data depend on the parameterization selected and the assumptions of the simulation model. Moreover, these dataset may constitute a partial representation of reality as the complexity of RNA-seq data is hard to mimic. Typical gold-standard (4) for gene expression are qPCR data (Bullard et al., 2010; Rapaport et al., 2013). However, analysis models for qPCR data should themselves be validated (Steibel et al., 2009). The use of plasmodes (5) is another appropriate procedure that can be applied to validate a statistical method. This approach aims at generating datasets that preserve the characteristics of experimental data with the benefit of knowing the true status as it happens with simulated data.

A plasmode is a dataset obtained from experimental data but for which some truth is known (Mehta et al., 2004). Plasmodes have been applied in microarrays (Gadbury et al., 2008), admixture estimation methodologies (Vaughan et al., 2009) and qPCR (Steibel et al., 2009). This procedure has not been extensively applied in RNA-seq since it requires large sets of raw data with an accurate description of the experimental conditions under which they were obtained. This information is essential to accurately develop plasmodes under null and alternative hypotheses. Only recently, an initiative has provided a repository with ready-to-use databases from RNA-seq studies (Frazee et al., 2011).

Processed data obtained from RNA-seq experiments are essentially counts that in the simplest model represent total number of reads mapping to a region in a reference genome or transcriptome. A comprehensive comparison of stochastic models that have been proposed is presented in Pachter (2011). Although different discrete distributions such as binomial, multinomial, beta-binomial, Poisson, and negative binomial, have been proposed to model RNA-seq data, Poisson and negative binomial are the most implemented ones in RNA-seq analysis software. A simple Poisson model seems appropriate when the experiment includes only technical replicates from a single source of RNA (Marioni et al., 2008). In practice, however, due to extra sources of variation, the observed dispersion is larger than the expected for a simple Poisson distribution and to correctly account for over-dispersion, generalized Poisson (GPseq) (Srivastava and Chen, 2010), mixed Poisson (TSPM) (Auer and Doerge, 2011), Poisson log-linear (PoissonSeq) (Li et al., 2012) and negative binomial (edgeR, DESeq, baySeq, NBPSeq) (Anders and Huber, 2010; Hardcastle and Kelly, 2010; Robinson et al., 2010; Di et al., 2011) are used instead. Regardless of the model, calculating dispersion parameters requires special statistical and numerical approaches due to the small sample sizes and large number of responses used in RNA-seq studies. In particular, borrowing information across transcripts when estimating model parameters, as used in microarrays (Smyth, 2004; Cui et al., 2005), has been also proposed for RNA-seq (Robinson and Smyth, 2008; Anders and Huber, 2010; Zhou et al., 2011). Another challenging issue for these statistical analysis models, is the ability to handle different experimental sources of variation. Most of the models allow fitting simple effect models and pair-wise comparison between treatments but only a few allow multiple factors (McCarthy et al., 2012). Currently, to the best of our knowledge, there is only one available model that can fit random effects (Van De Wiel et al., 2013). Methods that can accommodate complex hierarchical designs and provide more powerful tests to detect differentially expressed transcripts are under actively research. On the other hand, microarray analysis models and software usually assume a Gaussian distribution for response variables, but they accommodate fixed and random effects in a straightforward manner (Cui et al., 2005; Rosa et al., 2005). Consequently, an alternative to model counts in RNA-seq experiments is to transform counts and use Gaussian models (Langmead et al., 2010; Smyth et al., 2012).

In any case, given the multitude of available statistical models and the complexity of experimental design of many gene expression studies, researchers often find themselves having to decide between competing models and analysis program. In other cases, although a researcher may have an a priori designated software and model for RNA-seq data analysis, the question is if the fitted model is producing sound inferences.

In this paper, we present and apply a methodology for evaluating statistical methods for RNA-seq experiments by combining results from computer simulations and plasmodes. We follow the epistemological guidelines stated in Mehta et al. (2006) for high-dimensional biology and provide a general framework that can be adapted to different experimental conditions.

## Materials and methods

### Simulations

Simulated datasets were created conditional on estimated parameter values and results that had been previously obtained (Ernst et al., 2011). The data consisted on read counts from an RNA-seq experiment based on a developmental expression study (Sollero et al., 2011). Experimental and alignment protocols are described in the supplemental material (Supplementary Figure 1). Estimations for parameters μ_*i*_ and σ^2^ were obtained by fitting generalized linear Poisson models with log-library size as an offset variable using function lmer (Bates et al., 2013) from R (R Core Team, 2013).

Equation [1] represents the generalized linear model used to generate the simulated datasets:

(1) $$\{\begin{array}{c} \;y_{ij}\sim \text{Poisson}\left(\lambda_{ij}\right)\\ \text{log}\left(\lambda_{ij}\right)=O_{ij}+\mu_{i}+e_{ij}\\ \;e_{ij}\sim N\left(0,\sigma^{2}\right) \end{array}$$

where *y*_*ij*_ is the read count for a particular transcript in treatment *i* and sample *j*, *O*_*ij*_ is a known off-set value (in this case the total library size), μ_*i*_ is the group mean, *e*_*ij*_ is a sample-specific residual. The transcript sub-index (g) was omitted for convenience.

**Figure 1:**
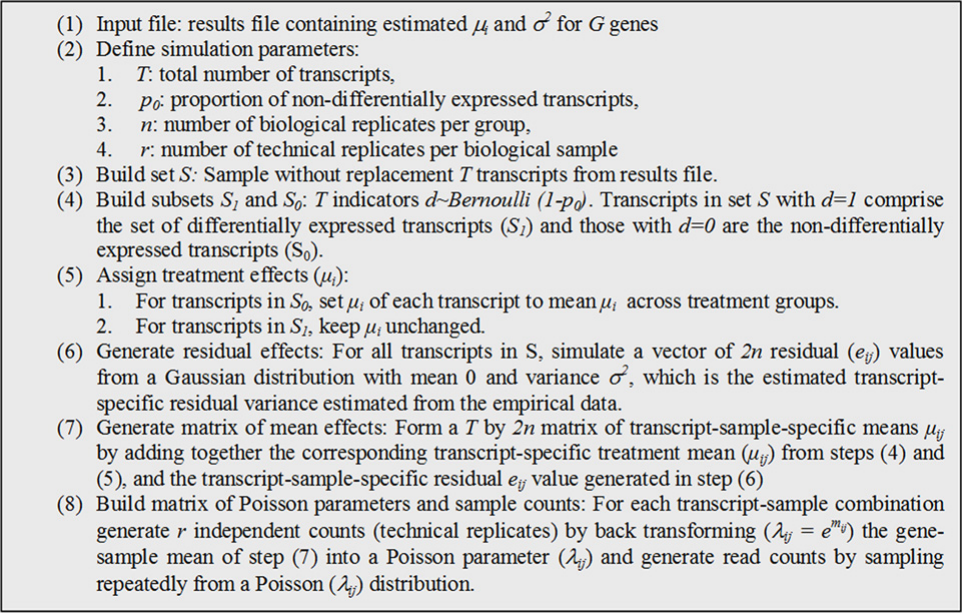
**Algorithm used to simulate counts from existing estimates of model parameters**.

Given estimates of parameters from equation [1] for transcripts, we simulated read counts by following the algorithm described in Figure 1. The output from such procedure consisted of a matrix of counts of size *T* by *2nr* with a known proportion (*p*_0_) of differentially expressed transcripts and known group effects (μ_*i*_). Treatment is represented in this matrix by *nr* columns, but with only *n* independent (biological) replicates. While this simulation is not based on the negative binomial distribution, it is still an over-dispersed Poisson process commonly used to simulate RNA-seq counts (Blekhman et al., 2010; Auer and Doerge, 2011; Hu et al., 2011). The resulting over-dispersed Poisson counts have means, variances, and treatment effects sampled from those estimated from experimental data. The procedure can be repeated K times to produce several simulated datasets.

We set *K* = 1000 and *T* = 5000, producing 1000 simulated datasets with 5000 transcripts each. Noteworthy, when sampling transcripts in S, it is assumed that all transcripts are differentially expressed (no significance testing is performed). But subsequently, the mean treatment differences (in the log-scale) are zeroed out if the transcripts are assigned to *S*_0_. For transcripts assigned to *S*_1_, mean differences are kept unchanged; consequently *S*_1_ includes a whole distribution of treatment effects from very small to large according to the distribution of estimated from the experimental data.

#### Replication scenarios

We simulated nine scenarios by combining three levels of biological replication (*n* = 3, 5, 10) and three levels of technical replication (*r* = 1, 3, 5). The proportion of differentially expressed transcripts was set to 0.1.

### Plasmodes

In contrast to simulation datasets based on Equation [1], we generated plasmode datasets not based on any model. Plasmodes were generated using data available in the online resource ReCount (Frazee et al., 2011). From the whole collection of analysis-ready datasets, we chose to work with two RNA-seq experiments to illustrate the generation of (1) a null dataset, where there are no obvious systematic effects that explain variance in gene expression and, (2) a dataset with treatment and block effects.

#### Null dataset (Cheung)

The data originated in a study of immortalized B-cells from 41 (17 females and 24 males) unrelated CEPH (Center d' Etudes du Polimorphisme Humain) grandparents (Cheung et al., 2010). The samples were sequenced using the Illumina Genome Analyzer. To generate a plasmode dataset, we selected the 21 samples from male individuals that were represented with only one technical replicate. The resulting gene expression data exhibits extensive variation that cannot be attributed to any systematic factor (Figure 3A). Any random partition of the dataset into two (or more) categories should shield a null dataset where no differential expression is expected beyond the normal sample-to-sample variation. Consequently this dataset lends itself to create plasmodes to evaluate statistical properties of analysis models under the null hypothesis.

**Figure 2:**
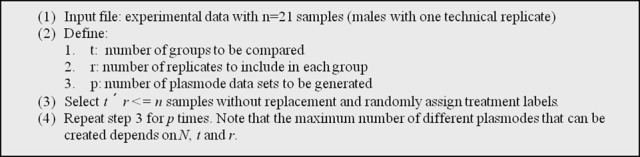
**Algorithm used to generate plasmode datasets with no differentially expressed transcripts under a model with one classification variable**.

To generate null datasets, we proceeded as explained in Figure 2. Using *n* = 21 samples from males, we generated *p* = 10 plasmodes each with *t* = 2 groups and *r* = 10 biological replicates in each group.

Notice that not parametric model is used at any time. Plasmodes are constructed by reshuffling data and assigning an arbitrary treatment label. In this way overall distribution and gene-to-gene correlations remain unchanged with respect to real data.

#### De dataset (Bottomly)

**Figure 3:**
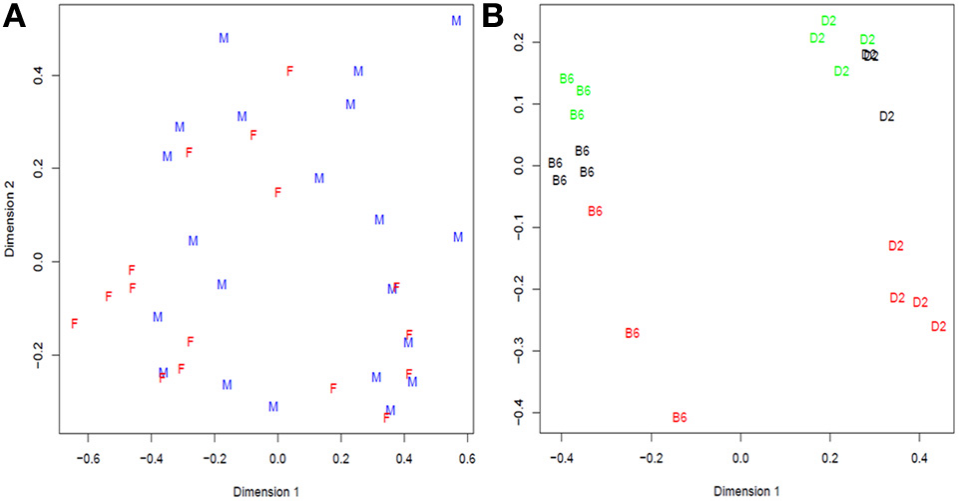
**Multidimensional scaling analysis of: (A) Cheung samples: F = Females and M = males; (B) Bottomly samples: labels correspond to strain (treatment) B6 = C57BL/6J, D2 = DBA/2J, and colors to flowcell number (block): red = 4, black = 6, and green = 7**. In Cheung dataset there is not clear distinction between females and males while in Bottomly samples are first grouped in two large groups corresponding to strain B6 and D2 and then in subgroups consistent with flowcell number.

In Bottomly et al. (2011), the authors arranged 21 samples from two inbred mouse strains (B6 and D2; *n* for B6 = 10, *n* for D2 = 11) on 21 lanes of three Illumina GAIIx flowcells and they analyzed the RNA-seq reads with a simple one-way classification (strain) model. We performed descriptive analysis of gene expression data and found that not only strain but also the experiment number (flowcell) explained a large amount of the variation (Figure 3B). For example, the first principal dimension clearly divides samples from each strain, but the second principal dimension shows substantial variation between flowcells, especially flowcell 4 (red) vs. the other two.

**Figure 4:**
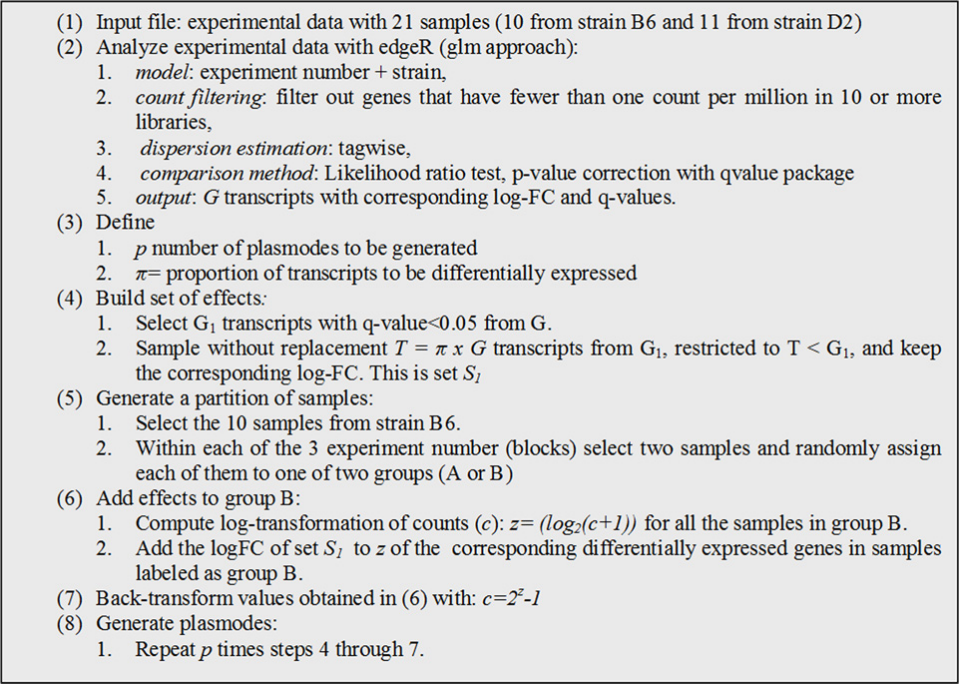
**Algorithm used to generate plasmode datasets with differentially expressed transcripts under a model with two classification variables (block + treatment)**.

Consequently, we blocked by experiment and used edgeR to fit a model with strain and experiment as fixed effect, resulting in a large number of putatively differentially expressed genes (Supplementary Figure 2). Due to a strong experiment effect, we decided to conduct randomization for plasmode construction within experiment number as detailed in Figure 4.

We generated 10 plasmodes executing step 4–7 with *p* = 10 and π = 0.20. Notice that in step 3, we used edgeR to obtain a list of DE genes (set G) to build a plasmode with some genes under alternative hypothesis but any other statistical software can be used with the only requirement of defining a sufficient small *q-value* threshold. After genes are selected no model is used at any time. Similar to the previous section plasmodes are constructed reshuffling data, but in this case and effect estimated from real data is added to selected genes. Again, we expect that this procedure yields plasmodes with identical distribution to real data for non-differentially expressed genes and with comparable effect sizes for differentially expressed genes.

### Comparison of alternative analysis tools for evaluating differential expression

To illustrate the use of simulated datasets and plasmodes we compared three R (R Core Team, 2013) packages from Bioconductor (Gentleman et al., 2004). Two of them, edgeR and DESeq, were designed specifically for statistical analyses of RNA-seq experiments while the third one, MAANOVA (Cui et al., 2005), was originally conceived for analyzing microarray experiments. As mentioned before, MAANOVA has the ability of fitting hierarchical models that can better accommodate complex experimental design assumptions. However, such flexibility comes at the price of assuming a Gaussian distribution. Data transformation and use of permutation to set significance thresholds can help alleviate these limitations, but its performance may still be contingent upon sample size and total read counts per transcript. Consequently, we included MAANOVA in this study and compare it to two well-established packages for RNA-seq analysis.

#### Filtering and normalization

A double filtering criterion was applied to all datasets previous to normalization and statistical analysis. Transcripts with 2 or more reads per million in at least as many libraries as number or biological replicates were kept in the analysis. In the simulation study, technical replicates were summed up before filtering. Consequently, the technical replicate level only represents increased sequencing depth.

Normalization aimed at accounting for differences in library size and composition not attributable to treatments. To conduct the analysis with edgeR, data were normalized using the scaling method proposed by Robinson and Oshlack (2010) and the logarithm of the resulting effective library size were used by default as offsets in the model.

Analyses with DESeq were performed on counts previously normalized by function estimateSizeFactors. According to Anders and Huber (2010), this normalization method is similar to the one proposed by Robinson and Oshlack (Robinson and Oshlack, 2010) in edgeR, and it is the recommended procedure by the authors of DESeq.

Normalized values to use in MAANOVA were obtained with function voom() of the limma package (Smyth, 2005). The process, analogous to the one proposed in (Smyth et al., 2012), included adjustment for compositional structure using function calcNormFactors() of edgeR and transformation to log2-counts per million.

#### Differential expression analysis

***edgeR.*** Differential expression was tested by likelihood ratio tests using the generalized linear model functionality and estimating tagwise dispersions.

***DESeq.*** To look for differentially expressed genes, function nbinomGLMTest was applied using the dispersion estimates generated by function estimateDispersions.

***MAANOVA.*** In the linear model fit by MAANOVA lane was treated as a fixed array effect of a single-color microarray. Differential expression analysis was performed using both, moderated *F*-test (*F*s) and transcript by transcript *F*-test (F1). Significance was assessed using 100 sample permutations (Yang and Churchill, 2007).

***Multiple comparisons.*** It is recognized that correction of *p*-values when making multiple comparisons is essential in high throughput differential expression analyses (Storey and Tibshirani, 2003). The most common procedure used is the computation of the false discovery rate or FDR (Benjamini and Hochberg, 1995). Properties of methods to estimate FDR rely heavily on the distribution of *p*-values (Li et al., 2012). In this case we did not aim at selecting individual differentially expressed genes or gene sets but we aimed at studying the properties of tests in terms of type I and type II error rates. Consequently, we concentrate on comparison of nominal and empirical type I and type II error rates without applying multiple correction and we discuss how departures of assumed values can further affect decisions when applying *p*-value corrections.

#### Evaluating and comparing results from alternative analysis packages

To compare performances of derived tests in terms of power and type I error rates, we generated receiver operator characteristic (ROC) curves by computing true positive rate (TPR) and false positive rate (FPR) at given significance thresholds. The TPR was calculated as the proportion of true positives (TP) over the total number of simulated differentially expressed transcripts (S_1_), while the FPR was calculated as the proportion of false positives (FP) over the total number of transcripts simulated with no differential expression (S_0_). See Table 1 for details.

**Table 1:** **Classification rule to compute false and true positive rates**.

	**Transcripts simulated with not differential expression**	**Transcripts simulated with differential expression**	**Total**
Transcripts not declared significant	TN	FN	R_0_
Transcripts declared significant	FP	TP	R_1_
Total	#S_0_	#S_1_	G

FP, number of false positives (transcripts in S_0_ set declared differentially expressed); TP, number of true positives (transcripts in S_1_ declared differentially expressed); FPR, false positive rate = FP/#S_0_; TPR, true positive rate = TP/#S_1_.

Finally, distributions of *p*-values were compared by quantile-to-quantile plots and histograms.

Analyses were performed at the Michigan State University High Performance Computing Center facilities using R (version 2.15.1), edgeR (version 3.0.8.4.6), limma (version 3.14.4), DESeq (version 1.10.1) and MAANOVA (version 1.28.0).

## Results

### Simulations

**Figure 5:**
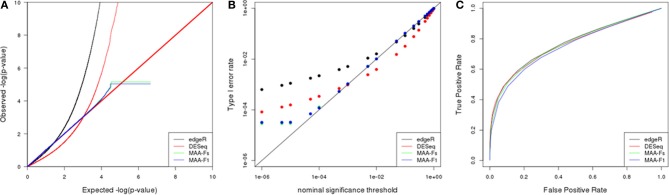
**Simulation results from a scenario with 3 biological replicates: (A) Q–Q uniform plot of non differentially expressed transcripts, (B) type I error rate vs. nominal significance values, and (C) ROC curves**. Models: (1) edgeR (blue), (2) DESeq (red), (3) MAA-Fs: MAANOVA *F*s moderated test using permutation (green), and (4) MAA-F1: MAANOVA F1 transcript by transcript test using permutation (blue).

Figure 5 shows results obtained for a simulation with 3 biological replicates and 1 technical replicate. Similar results were found in other simulated scenarios (data not shown).

The Q-Q plot in Figure 5 allows to evaluate the fit of observed *p*-values to the uniform (0,1) distribution expected under null hypothesis (Leek and Storey, 2011). *P*-values corresponding to MAANOVA showed a more characteristic pattern whereas edgeR and DESeq presented significant departures from such distribution. Furthermore, the logarithmic scale allows to easily inspect the behavior of very small *p*-values. DESeq presented larger *p*-values than expected up to a cutoff of 0.001, while the opposite pattern occur for *p*-values smaller than 0.001. Both MAANOVA approaches presented a close to expected pattern with a small deviation for *p*-values smaller than 0.0001. To compute the logarithm, all *p*-values equal to zero were replaced by the minimun observed *p*-value and thus generated the plateau at the end of the distributions of MAANOVA results. In addition, quantile-to-quantile plots allowed us to select Fs and F1 tests computed with permutation against the tabulated approach (Figures 8A,B). An alternative representation of *p*-value distribution using histograms is presented in the supplemental material (Supplementary Figure 3).

In concordance with the observed *p*-value distributions, the realized type I error rates levels for DESeq and edgeR were much different than expected in comparison with MAANOVA approaches (Figure 5B). All the packages presented higher realized significance levels when evaluated at nominal values bellow 0.01, with edgeR being the most liberal, and MAANOVA the least deviated from nominal values.

ROC curves had similar patterns for each of the nine simulated scenarios. Power improved at a given FPR as the number of technical and/or biological replicates increased. In the scenario with 3 biological replicates, the enhancement in power when adding technical replicates seems to be particularly greater than in a scenario with 5 or 10 biological replicates (data not shown). In the case with 3 biological replicates and 1 technical replicate (Figure 5C), edgeR and DEseq had similar power while the MAANOVA analyses reported less power.

### Plasmodes

#### Null dataset (Cheung)

**Figure 6:**
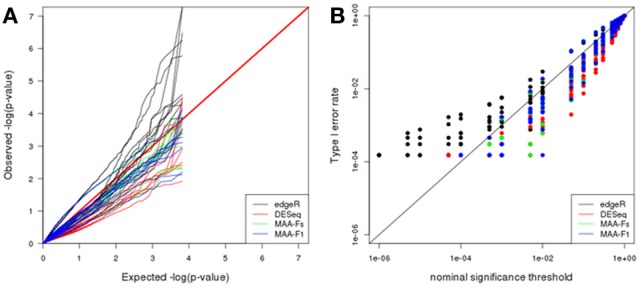
**Plasmode results from Cheung dataset: (A) Q-Q uniform plot of non-differentially expressed transcripts, (B) type I error rate vs. nominal significance values**. Models: (1) edgeR (blue), (2) DESeq (red), (3) MAA-Fs: MAANOVA Fs moderated test using permutation (green), and (4) MAA-F1: MAANOVA F1 transcript by transcript test using permutation (blue).

Q-Q plot in Figure 6A shows the adequacy of *p*-values to the uniform distribution for each of the plasmode datasets analyzed with the different models. All the models presented large dispersions with some cases being close to the expected values and some being far apart. In particular, edgeR results tend to be above the identity line which means that observed *p*-values are smaller than expected. On the contrary, DESeq and both MAANOVA tests tend to have a more conservative behavior as they presented larger observed *p*-values than expected. See also Figure 6B where edgeR presented inflated type I error rates for nominal significance threshold smaller than 0.01.

#### Bottomly

**Figure 7:**
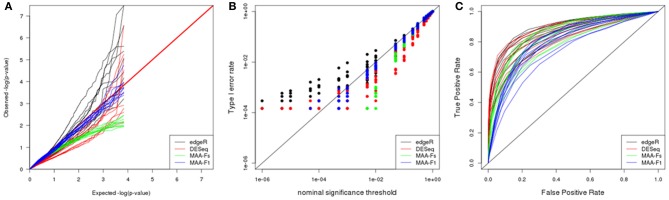
**Plasmode results from Bottomly dataset: (A) Q-Q uniform plot of non-differentially expressed transcripts, (B) type I error rate vs. nominal significance values, and (C) ROC curves**. Models: (1) edgeR (blue), (2) DESeq (red), (3) MAA-Fs: MAANOVA Fs moderated test with permutation (green), and (4) MAA-F1: MAANOVA F1 transcript by transcript test tabulated (blue).

The *p*-value distributions (Figure 7A) presented similar dispersion patterns to the one observed in the plasmodes generated from Cheung dataset utilizing edgeR and DESeq. However, *p*-value distributions for MAANOVA tests were more homogeneous across datasets with the *p*-values from F1 test tabulated approach being closer to the expected values under uniform distribution.

ROC curves for DESeq and edgeR were analogous after adjusting for type I error rates. Besides, both programs reported higher power than analysis performed with MAANOVA (Figure 7C).

**Figure 8:**
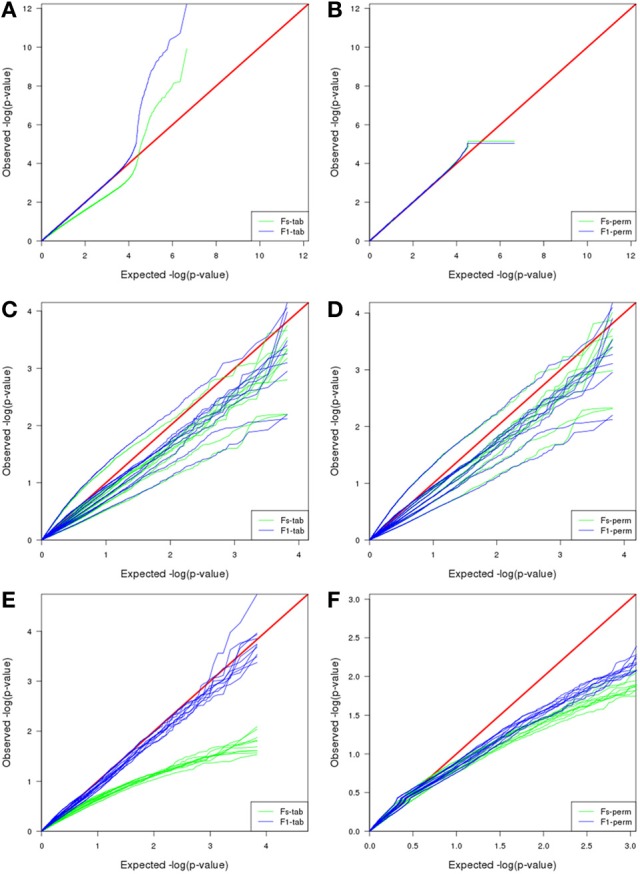
**Comparison of MAANOVA's *p*-value results of non-differentially expressed transcripts using *F*s moderated test and F1 transcript by transcript test, with a tabulated (left) or permutation (right) approach**. In the simulated dataset **(A,B)** the permutation approach presented a more characteristic uniform distribution, the plateau at the end is caused by the replacement of zeroes by the minimum observed *p*-value when computing logarithm. Plasmodes generated from Cheung dataset, presented similar patterns either using a tabulated or a permutation approach **(C,D)**. Plasmodes generated from Bottomly presented better patterns for *F*s with permutation and F1 with tabulated approach **(E,F)**.

Interestingly, and opposite to previous datasets, the best *F*-test to apply when using MAANOVA was F1 with tabulated *F*-values. Compare the proximity to the red line in Figure 8E in contrast to the pattern in Figure 8F.

## Discussion

Validating and comparing methods to analyze RNA-seq data is essential for providing powerful statistical packages that can detect differentially expressed genes in downstream analyses (Robles et al., 2012). In this paper we illustrate how to utilize plasmode datasets in combination with simulations to evaluate analysis methods more comprehensively.

Parametric simulations can benefit a particular model depending on the distribution and specifications used to generate the dataset. For example, it can be argued that in our simulation study, edgeR and DESeq resulted too liberal compared to MAANOVA due to the additive generalized Poisson model that was used to simulate the dataset. However, results from two independent plasmode datasets, generated without using specific parametric models, confirmed the same behavior (Figures 6B, 7B). Moreover, a common problem of parametric simulations is that genes are simulated independently. Such misspecification is overcome in plasmode datasets where the residual correlation structure among genes after adjusting for systematic effects is preserved with respect to the original dataset.

Exploring the joint null distribution of *p*-values for a particular test helps to determine the adequacy of a model and to decide the best method to correct for multiple comparisons, and doing so requires generation of multiple accurate high-dimensional datasets (Leek and Storey, 2011). For example, we compared null *p*-value distribution obtained for the two types of MAANOVA *F*-tests (*F*s or F1) combined with two methods to compute the *p*-values (tabulated F or permutation). The choice of the best combination varies for each dataset: In the simulation study, either *F*s or F1 using permutation provide a *p*-value distribution closed to a uniform distribution while none of the *F*-tests using tabulated values provide a reasonable distribution (Figures 8A,B). Plasmode generated from Cheung datasets presented similar patterns for all the combinations (Figures 8C,D), then *F*s and F1 using permutation were chosen as suggested by Cui et al. (2005). Conversely, in the analysis of plasmodes generated from Bottomly datasets, F1 test using tabulated *F*-values was the best approach (Figures 8E,F). According to Cui et al. (2005), the F1-test for a fixed effect model has a standard *F* distribution and critical values could be obtained from *F*-tables. These results are important because typical correction by FDR as proposed by Benjamini and Hochberg (1995) may not be appropriate if the underlying uniform distribution is not supported. Other strategies have been adapted from Storey (2002) to estimate FDR for RNA-seq data and which correction should be applied is a topic of research (Li et al., 2012). All in all, these results emphasize the need to validate methods under realistic conditions and carefully selecting a base dataset for a plasmode where total sample size and sequencing depth (magnitude of counts) are considered.

In addition to the base dataset used to build a plasmode, the specific algorithm for plasmode generation should vary according to the objective of the study. Gadbury et al. (2008) presented an algorithm that generates the partition of the samples in two groups and repeatedly samples different effect sets to be added to that unique partition. In this work, we propose to make several partitions from the original set of samples and add a set of effect in each case (Figure 4). This approach constitutes a way to incorporate valuable information on biological variation. For example, one can easily study the dispersion of patterns in the Q-Q plots or ROC curves. Alternatively, both approaches, Gadbury et al. (2008) and the one presented in this paper, can be combined to study the influence of different sets of genes as well as sample variability.

Moreover, the construction of a plasmode must consider all the experimental conditions under which the base data were collected. Treatment and block effects may be easily identified from the experimental design but further restrictions in randomization (flowcell, lane, time) or technical issues (operator, use of technical replicates) may arise only from inspecting protocol details and applying explorative statistical analyses. For instance, descriptive analysis of the Cheung dataset and visualization of samples using multidimensional scaling analysis (Figure 3A) suggested that no specific effects were present in the data structure; therefore we used it as an example to build a null plasmode. However, the same procedure applied to the Bottomly dataset indicated that not only the main strain, but also a characteristic effect due to flowcell number was an important source of variation (Figure 3B). Consequently, strain and block (flowcell) were considered in two parts of the plasmode generation algorithm: firstly, when defining the model to select the effects (step 2 in Figure 4), and secondly, when partitioning samples within each flowcell (step 5 in Figure 4). These considerations allowed us to generate appropriate null and alternative datasets. A similar process should be followed with any new dataset plausible of being used as a base for plasmode generation.

We used the plasmodes and simulated data to illustrate the selection of optimal differential expression analysis strategies. To this end, we focused in comparing true and false positive rates of tests to assess type I error rates and power. While it was not our objective to perform a comprehensive evaluation of analysis protocols for RNA-seq data analysis, we did want to include two broad types of methods: (1) those directly tailored to count data by using negative binomial distributions (DESeq, EdgeR) or (2) a Gaussian model after transformation (MAANOVA). We found that edgeR and DESeq incur in inflated type I error rates for small significance levels (Figures 5B, 6B, 7B) while MAANOVA's *p*-values tend to be closer to the nominal significance levels. Admittedly, after adjusting for type I error rates, power was similar for edgeR and DESeq and higher than that from MAANOVA (Figure 7C). However, in a real data scenario, adjusting is not possible because the true status is unknown.

These results emphasize the fact that RNA-seq data are complex and to decide what method to use may be experiment-specific due to the unknown distributions of expression levels. Plasmode may contribute to decide which method to choose by using a similar pre-existing dataset and comparing results. It is critical to select a dataset that has a complete description of the experimental design and detailed protocols of how the data were obtained. Using this information, it is possible to design proper null and alternative datasets. For example, it was easy to find a set of differentially expressed genes in the mouse dataset that studied two inbred lines. Contrarily, in the human dataset, it was not possible to explain the variation in expression only as a consequence of gender effects. The human subjects came from an outbred population and factors such as age, weight, or other characteristics could have explained differences in gene expression. Granted, any of the mentioned effects could have been included in the model if the information was available. The promising results obtained from this approach, emphasize the need of promoting and improving systematic data sharing across the research community to facilitate plasmode building.

Finally, the flexibility of plasmode construction allows comparing model tuning selection for downstream analysis but also upstream analysis, as normalization procedures or alignment pipelines, could be contrasted. Future uses of plasmodes could be: comparison of alignment programs for a given statistical analysis model or even exploring interaction of statistical model and read processing protocols to find optimal combined pipelines for data processing “from reads-to-*p*-values.”

### Conflict of interest statement

The authors declare that the research was conducted in the absence of any commercial or financial relationships that could be construed as a potential conflict of interest.
