# Supplementary material for: Evaluating statistical analysis models for RNA sequencing experiments
Source: Front Genet. 2013 Sep 17;4:178. doi: 10.3389/fgene.2013.00178 (PMC3775431; doi:10.3389/fgene.2013.00178)
Supplement: Supplementary file 1 [file DataSheet1.DOCX]

**Supplemental Material**

Reeb, P.D, Steibel, J.P. Evaluating statistical analysis models for RNA sequencing experiments.

**RNA-seq data processing**

In a previous experiment, we used the Pigoligoarray for transcriptional profiling of developing pig skeletal muscle, and results for a study comparing transcript profiles of *longisimus dorsi* muscle from fetuses at 40 and 70 d of gestation in two different breed (Sollero et al., 2011). For this study, we used the same RNA samples from one of the breed types profiled with the Pigoligoarray (n = 3 for each developmental age) with deep sequencing technology (Illumina GAIIx) to obtain 50nt paired end reads from 6 libraries (2 conditions, 3 bio-replicates each). The processing steps are described in Figure 1 Supp.

1. Filter passing read pairs were aligned to the S. scrofa reference genome (Sscrofa9, April 2009, Ensemble release 61) using the spliced RNA aware aligner, TopHat. Reads from each library were aligned separately. The reference gene annotation (same version as above) was provided to TopHat to provided information about predicted splice junctions, but TopHat also predicts novel splice junctions.

2. Novel splice junctions predicted from each of the 6 libraries were combined with the splice annotations from the reference to create a single, non-redundant set of predicted splice sites. TopHat is better able to map spliced reads if a list of potential junctions is provided as input.

3. Each library was aligned to the reference a second time, providing the non-redundant set of potential splice sites as input.

4. The alignments produced from each library were used as input to Cufflinks. Cufflinks was used to examine RNA-Seq alignments and to generate a set of predicted transcripts based on assembly of overlapping reads.

5. The transcript models generated by Cufflinks for each library were combined into a single, non-redundant set of transcript/gene models with Cuffcompare. The reference annotation was also provided to Cuffcompare to associate the predicted gene models with their most likely reference model.

6. The models generated by Cuffcompare are filtered to remove those models with little support from the underlying read alignments. Specifically, if aligned reads are observed in only one of the six libraries, that model is removed from the final set.

7. The alignments for each library produced during the second round of TopHat (step 3) and the curated set of gene models (step 6) were used as input to htseq-count. This program compared a set of alignments to an annotation file and reported the number of fragments uniquely aligned to each gene in the annotation. The models generated by Cuffcompare may have multiple transcripts modeled for a particular gene but htseq-count only reports the total fragments for a gene.

**Figure 1 Supp.** Steps used to process reads to obtain matrix of counts

**References**

Sollero, B. P., Guimarães, S. E. F., Rilington, V. D., Tempelman, R. J., Raney, N. E., Steibel, J. P., Guimarães, J. D., Lopes, P. S., Lopes, M. S., and Ernst, C. W. (2011). Transcriptional profiling during foetal skeletal muscle development of Piau and Yorkshire–Landrace cross-bred pigs. *Animal Genetics* 42, 600–612.


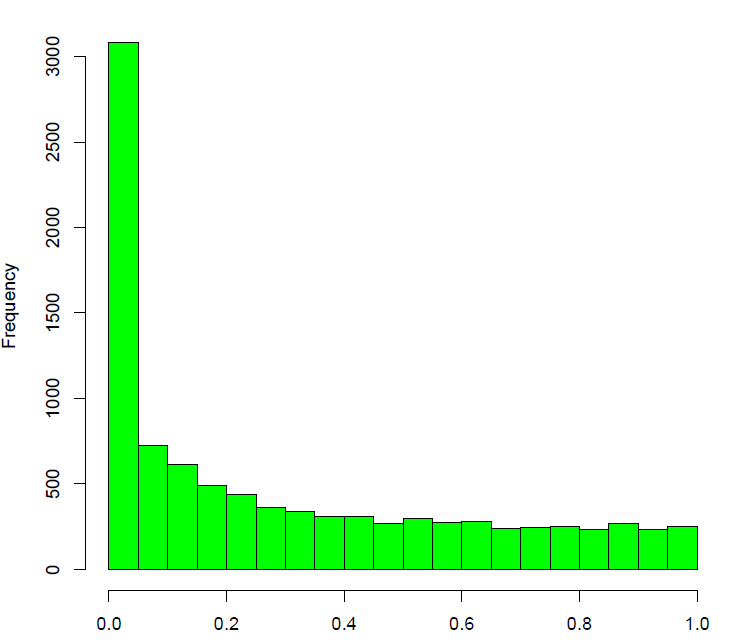


**Figure 2 Supp.** P-value distribution of differential expression analysis performed with edgeR for Bottomly data using a model with block and treatment fixed effects.

**Figure 3 Supp.** P-value distribution of non differentially expressed transcripts for a simulated scenario with 3 biological replicates using: 1) edgeR, 2) DESeq, 3) MAA-Fs: MAANOVA Fs moderated test (permutation), and 4) MAA-F1: MAANOVA F1 transcript by transcript test (permutation).


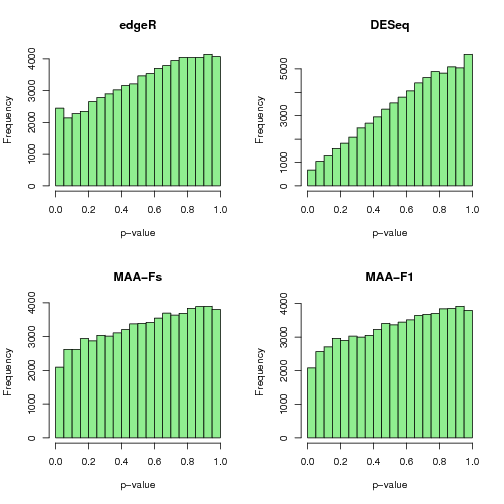


**Figure 4 Supp.** P-value distribution of non differentially expressed transcripts for plasmodes generated from Cheung dataset using: 1) edgeR, 2) DESeq, 3) MAA-Fs: MAANOVA Fs moderated test (permutation), and 4) MAA-F1: MAANOVA F1 transcript by transcript test (permutation).


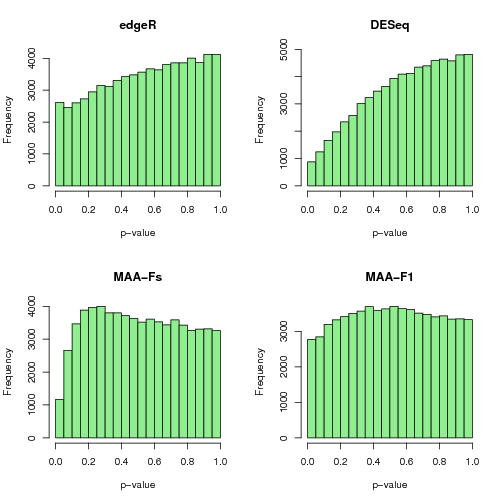


**Figure 5 Supp.** P-value distribution of non differentially expressed transcripts for plasmodes generated from Bottomly dataset using: 1) edgeR, 2) DESeq, 3) MAA-Fs: MAANOVA Fs moderated test (permutation), and 4) MAA-F1: MAANOVA F1 transcript by transcript test (tabulated).
